# Starch Acetate Grafted to MXene Composite Surpasses Room Temperature Liquid Electrolyte Performance for All‐Solid‐State Lithium‐Ion Batteries

**DOI:** 10.1002/advs.202503285

**Published:** 2025-06-19

**Authors:** Saeed Hadad, Mahtab Hamrahjoo, Homayun Khezraqa, Marzieh Golshan, Zhaohui Wang, Mehdi Salami‐Kalajahi

**Affiliations:** ^1^ Faculty of Polymer Engineering Sahand University of Technology P.O. Box 51335‐1996 Tabriz Iran; ^2^ Institute of Polymeric Materials Sahand University of Technology P.O. Box 51335‐1996 Tabriz Iran; ^3^ College of Materials Science and Engineering Hunan University Changsha 410082 China

**Keywords:** all‐solid‐state lithium‐ion‐batteries, click chemistry, MXene quantum dots, natural polymers, solid polymer electrolytes, starch acetate

## Abstract

A groundbreaking solid polymer electrolyte (SPE) design is reported that outperforms traditional liquid electrolytes in both performance and safety, while being environmentally benign. By leveraging click chemistry, starch acetate (SA) is integrated, a natural polymer itself capable of supporting superionic conductivity, with MXene quantum dots (MX‐QDs). While the composite electrolyte is electrically insulating, the electrical conductivity of the MXene stabilizes the anionic species while also acting as a filler to boost mechanical properties. The optimized SPE composition, comprising 30 *wt*.% MX‐QDs, exhibits exceptional electrochemical characteristics: ionic conductivity of 14.8 mS cm^−1^, lithium cation transfer number of 0.91, and an electrochemical stability window of up to 5.2 V. Notably, this SPE demonstrates seamless compatibility with lithium metal anodes, enabling a solid‐state battery that retains 90% capacity over 1000 charge–discharge cycles. This innovative SPE design paves the way for the widespread adoption of solid‐state batteries in electric vehicles.

## Graphical abstract

By leveraging click chemistry, starch acetate is integrated with MXene quantum dots (MX‐QDs). While the composite electrolyte is electrically insulating, MXene stabilizes the anionic species while acting as a filler to boost mechanical properties. Optimized composition exhibits exceptional ionic conductivity of 14.8 mS cm^−1^, lithium cation transfer number of 0.91, and an electrochemical stability window of up to 5.2 V.

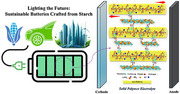

## 1 Introduction

Lithium‐ion batteries serve as efficient energy storage systems for sustainable sources, offering an alternative to fossil fuels and meeting the rising demand for electric vehicles and portable electronics.^[^ ^1^ ^]^ With the LIBs market projected to grow from US$44.5 billion to US$135.1 billion by 2031, their importance is undeniable.^[^ ^2^ ^]^ However, most commercial LIBs use toxic and flammable liquid electrolytes, posing safety risks and allowing dendrite growth.^[^ ^3^ ^]^ This accumulation of lithium on the anode surface reduces battery capacity over time, leading to short circuits and failure.^[^ ^4^ ^]^ Consequently, liquid electrolyte‐based metal‐ion batteries are not really sustainable due to limited metal sources, particularly lithium.

On the other hand, all‐solid‐state lithium‐ion batteries (ASSLIBs) have garnered significant academic and commercial interest due to their promise of improved safety, enabling higher capacity lithium (Li) metal anodes and potentially faster charging times.^[^ ^5^ ^]^ While solid‐state electrolytes (SSEs) based on sulfides and Li argyrodites are now often reported with conductivities >10 mS cm^−1^, solid polymer electrolytes (SPEs) have lagged behind, typically achieving < 1 mS cm^−1^ at room temperature (RT).^[^ ^6^, ^7^ ^]^ If SPEs could achieve similarly high conductivity and overcome challenges associated with low modulus, they might become the preferred choice for ASSLIBs due to their ease of processing, ability to make conformal interfacial contact with electrode materials, and their more elastic mechanical properties.^[^ ^6^, ^8^ ^]^

The ionic conductivity of SPEs is influenced by the presence of accessible electron donor atoms, such as oxygen or fluorine, that establish coordination with lithium cations (Li^+^) while electron acceptors help stabilize the associated anions. For this reason, synthetic polymers with high oxygen content, such as poly(ethylene oxide) (PEO),^[^ ^9^ ^]^ polycarbonate,^[^ ^10^ ^]^ and polyesters^[^ ^11^ ^]^ have been heavily researched. However, their conductivity is heavily impacted by the formation of crystalline domains that make coordination sites inaccessible and also act as barriers to ion transfer.^[^ ^8^ ^]^

Conversely, recent studies have shown that utilizing amorphous derivatives of natural polymers like cellulose, starch, and chitin^[^ ^12^ ^]^ can produce SPEs with high ionic conductivity and have achieved promising results in ASSLIBs. For instance, a blend of poly(polyethylene glycol methacrylate) p(PEGMA) and cellulose triacetate,^[^ ^13^ ^]^ PEO filled with bacterial cellulose fibre,^[^ ^14^ ^]^ cellulose‐based quasi‐solid,^[^ ^15^ ^]^ oxidized carboxymethyl cellulose,^[^ ^16^ ^]^ and starch acetate crosslinked with poly(vinyl alcohol)^[^ ^17^ ^]^ have achieved RT conductivities between 0.01 and 9 mS cm^−1^. However, the Li^+^ transference number in these studies has been relatively low compared to other SSEs, ranging from 0.43 to 0.82.

Additionally, incorporating an appropriate filler into the polymeric matrix can enhance both ionic conductivity and mechanical properties of the final SPE.^[^ ^18^ ^]^ Consequently, numerous studies have investigated the effects of various fillers, such as graphene‐based fillers ^[^ ^19^ ^]^ metal–organic frameworks (MOFs),^[^ ^20^ ^]^ and ceramics.^[^ ^21^, ^22^ ^]^ Incorporating fillers with electron acceptor atoms in their structure is a viable strategy to enhance ionic conductivity in polymer electrolyte systems. In these systems, the incomplete dissociation of Li salt leads to a series of equilibria, forming associated species: M^+^ + X^‐^ ⇋ MX, M^+^ + MX ⇋ M_2_X^+^, and X^‐^ + MX ⇋ MX_2_^‐^.^[^ ^23^ ^]^

Among all the charged species, only M^+^ (Li^+^) can participate in the reversible reduction‐oxidation reaction on an electrode surface.^[^ ^23^ ^]^ Hence, the use of such fillers stabilizes the anionic component (X^‐^) of the dissociated Li salt, preventing undesirable equilibria and allowing Li^+^ ions to remain active in the charge–discharge process. Notable examples of effective fillers include layered lithium montmorillonite,^[^ ^24^ ^]^ layered aluminosilicate halloysite nanotubes,^[^ ^25^ ^]^ and cationic MOFs.^[^ ^26^ ^]^ Despite demonstrating improvements, these studies have yet to achieve conductivity levels comparable to liquid electrolytes.

Herein, we aim to develop a novel SPE by integrating Ti_3_C_2_T_x_ MXene nanosheets into a starch acetate (SA) matrix. This hybrid system combines SA's eco‐friendly, cost‐effective, and oxygen‐rich backbone with the unique properties of MXene nanosheets. The titanium (Ti) atoms in MXene act as electron acceptors, while the multifunctional surfaces and oxygen‐containing groups facilitate Li^+^ coordination and crosslinking of SA. The fluorine (F) atoms on MXene reduce the dissociation energy of lithium salts, and the nanosheets’ mechanical properties enhance dendrite resistance.^[^ ^27^, ^28^ ^]^ To maximize interfacial stabilization of charged species, we used high weight percentages (10, 30, and 50%) of MXene quantum dots (MX‐QDs) and grafted SA chains onto MX‐QDs via copper‐catalyzed alkyne‐azide cycloaddition (CuAAC) click chemistry. This dense grafted layer prevents electrical contact between MXene sheets, enabling superionic conduction. Our optimized compositions achieved a record‐high room temperature conductivity of 14.8 mS cm^−1^ and a transference number of 0.91. This approach also increased the electrochemical stability window to 5.2 V, ensuring stable cycling with lithium metal and high‐voltage LiCoO_2_ cathodes.

## 2 Results and Discussion

### 2.1 Characterizations

**Figure 1:**
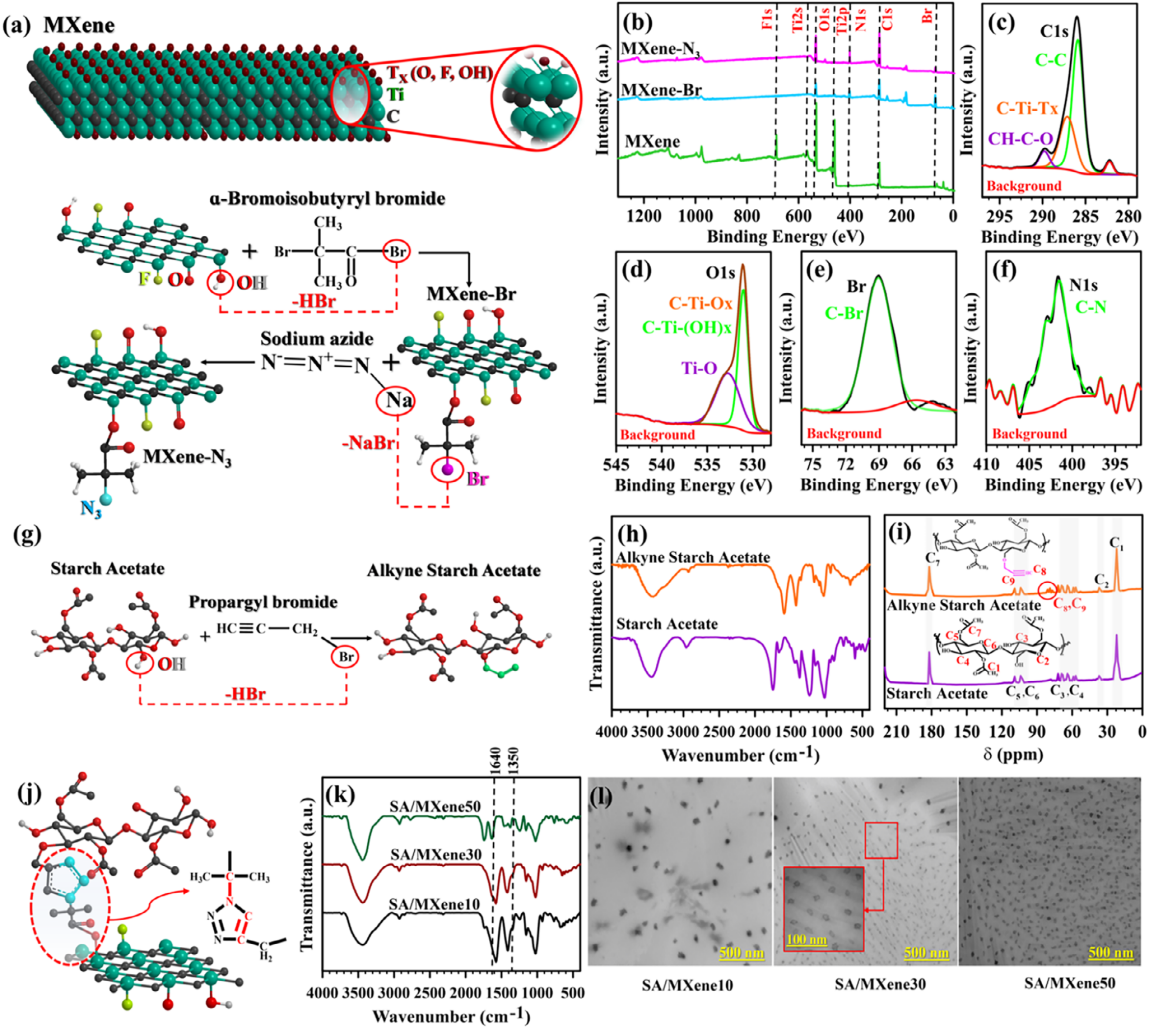
(a) Schematic representation of MXene nanosheets modification with BiBB and NaN_3_; (b to f) XPS results for MXene, MXene‐Br, and MXene‐N_3_ samples; (g) Schematic representation of SA modification with propargyl bromide; (h and i) FTIR and ^13^C NMR spectra of SA and Alkyne SA; (j) Schematic of CuAAC click reaction and triazole ring formation; and (k and l) FTIR spectra and TEM images of SA/MXene10, SA/MXene30, and SA/MXene50.

We first confirm the covalent modification of the MXene nanosheets with starch via click chemistry (**Figure** **1**a–f; Figure S1b–f, Supporting Information). The chemical grafting of SA chains onto the surface of the MX‐QDs is achieved through the CuAAC click reaction, which typically involves the coupling of alkyne (─C≡CH) and azide (N_3_) functionalities. Since neither MX‐QDs nor SA inherently possesses these functionalities, a two‐step modification process is employed to introduce N_3_ groups onto the surface of the MX‐QDs. Concurrently, alkyne groups are introduced onto the SA chains in a single step by reacting the OH groups with propargyl bromide. This strategic functionalization enables the successful CuAAC click reaction, facilitating the grafting of SA chains onto the MX‐QDs' surface.

FTIR spectra (Figure S1d, Supporting Information) show bands associated with the C─Br vibrational modes between 500 to 600 cm^−1^ for the MXene‐Br sample.^[^ ^29^ ^]^ This confirms the successful reaction of the hydroxyl (OH) groups on the MXene surface with BiBB to append bromine (Br) functionality. This is required to react with the azide functionality confirmed by FTIR to be on the MXene surface via observation of the azide symmetric and asymmetric absorbance visible near 2050–2200 cm^−1^ (a detailed analysis of the FTIR spectra is provided in Section S2.1, Supporting Information).^[^ ^30^ ^]^ To confirm FTIR results, X‐ray photoelectron spectroscopy (XPS) was performed (Figure 1b–f) after each modification step. The unmodified MXene nanosheets reveal signals for F 1s, O 1s, Ti 2p, and C 1s at their respective binding energies (682.75, 529.71, 459.52, and 285.91 eV, respectively).^[^ ^31^ ^]^ Following the modification of nanosheets with BiBB, a Br signal appears ≈69.73 eV. Further reaction with NaN₃ results in an N signal ≈402.21 eV in the MXene‐N₃ sample. Additionally, the deconvoluted signals for both Br (Figure 1e) and N (Figure 1f) indicate a single bond type and coordination environment for these atoms, confirming that the modifications were successfully carried out. X‐ray diffraction (XRD) patterns of all Ti₃C₂T_x_ MXene nanosheets, MXene‐Br, and MXene‐N₃ are presented in Figure S1e (Supporting Information). Reflections associated with Ti_3_C_2_T_x_ are observed at 2θ ≈ 9.7° (002 plane), 2θ ≈ 19.1° (004 plane), and 2θ ≈ 38.5°^[^ ^32^ ^]^; these peaks become broader with lower intensity after each modification step, particularly in the MXene‐N₃ sample. The full width at half maximum (FWHM) is observed to increase, suggesting a reduction in crystallite size, indicating better exfoliation or inhibition of restacking due to the modifications. While we observe reduced order, we do not see a significant shift to lower 2θ that would indicate an increase in d‐spacing. The addition of functional groups to the MXene surface may contribute to the broadening of the peaks rather than a noticeable shift. The conversion of MXene to MX‐QDs further decreases the intensity and broadens XRD peaks, likely due to the size reduction of the crystallites during the conversion process.^[^ ^33^ ^]^ This processing results in smaller quantum dots, which in turn affect the XRD patterns. Before and after comparisons show that the processing steps involved in converting MXene to MX‐QDs significantly contribute to the observed size shrinkage. Lastly, the addition of Br and N_3_ groups can also be detected in the TGA curves (Figure S1f, Supporting Information). While there is no degradation for Ti_3_C_2_T_x_ MXene nanosheets up to 600 °C, weight loss of 4.9 and 5.3 *wt*. % are observed for the MXene‐Br and MXene‐N_3_ samples, respectively (more detailed explanations of the TGA results can be found in Table S2, Supporting Information).

The introduction of the alkyne group to SA chains (Figure 1g) can be confirmed by both FTIR and carbon‐13 nuclear magnetic resonance (^13^C NMR) spectroscopy (Figure 1h,i, respectively). In the FTIR spectrum, the terminal alkyne (─C≡CH) stretching main peak appears ≈3300–3320 cm.^−1[^ ^34^ ^]^ However, this peak overlaps with the broad peak of hydroxyl (OH) groups on SA. Therefore, it is necessary to investigate the ^13^C NMR results to confirm the alkyne groups grafting; the emergence of a peak ≈75–80 ppm (C_8_ and C_9_) in the spectrum is evidence for the successful preparation of alkyne SA (detailed study of FTIR and ^13^C NMR results can be found in Section S2.1, Supporting Information).

The click reaction between the alkyne and azide groups results in a triazole ring (Figure 1j). To confirm the CuAAC reaction, FTIR spectroscopy is used. The visible absorbances (not overlapped with SA or MX‐QDs) of the final ring would be C─H bending (of triazole ring) ≈1350 cm^−1^ and C═C stretching ≈1640 cm^−1^, which are noted in Figure 1k.^[^ ^35^ ^]^ The primary goal of using the CuAAC click reaction was to ensure the proper dispersion of MX‐QDs without aggregation in the SA polymer matrix. The dispersion of MX‐QDs in the SA matrix was investigated by TEM as shown in Figure 1l. At all MX‐QD loadings (10, 30, and 50 *wt*.%), there is no aggregation, and the MX‐QDs are uniformly dispersed throughout the SA matrix. While the objective was to chemically attach SA chains to the MX‐QD surface via CuAAC click chemistry to ensure uniform dispersion and prevent aggregation, it is theoretically possible that some unreacted azide groups on the MXene surface or alkyne groups on the SA chains did not participate in the reaction. This could result in the presence of minimal amounts of free MXene or SA that are not chemically bonded within the composite. However, the TEM images demonstrate that any such free components are negligible and do not compromise the uniform dispersion, as no signs of aggregation are observed across all compositions. This is further corroborated by XRD patterns (Figure S1h, Supporting Information), which show no distinct MXene or SA phases, indicating that any unreacted material does not form separate, detectable domains.

Starch acetate chains are present both as chemically attached to the MX‐QD surface and as free chains within the matrix. An increase in MX‐QD content (from 10 to 50 *wt*.%) leads to a corresponding increase in the amount of chemically attached SA, reflecting the effectiveness of the click chemistry approach. The size of the MX‐QDs after dispersion in the SA matrix remains consistent with their original dimensions post‐synthesis, ≈10 nm (see Figure S1c, Supporting Information), suggesting that the modification and dispersion processes do not alter the MX‐QD size. In all TEM images, MX‐QDs are separated by distances of tens of nanometers, with no evidence of aggregation. This uniform separation ensures that no percolated electronically conductive network is formed, which could otherwise short‐circuit the SPE when used as a membrane separator between the anode and cathode. To validate this, a Four‐Probe DC test was conducted on all prepared polymer nanocomposites, revealing an electronic conductivity lower than 10⁻⁹ S cm^−1^ for all samples. This low conductivity confirms the absence of significant free MXene that could create conductive pathways, aligning with the TEM and XRD findings and supporting the structural integrity of the composite for its intended application.

Further characterization of the SA/MXene10, SA/MXene30, and SA/MXene50 samples, including differential scanning calorimetry (DSC) curves, XRD patterns, and TGA curves, can be observed in Figure S1g–I (Supporting Information). Both XRD patterns and DSC curves show the complete amorphous structures for all the prepared polymeric nanocomposites, which is a prerequisite for fast ion conduction in the starch system. Furthermore, no glass transition temperature (*T*_g_) step is observed in the DSC curves. While SA, with a degree of substitution of three, typically exhibits a *T*_g_ ≈150 °C,^[^ ^17^ ^]^ the absence of a *T*_g_ in our nanocomposites indicates the crosslinking effect of the MX‐QDs.^[^ ^36^ ^]^ The multifunctional surface of the MX‐QDs not only facilitates the attachment of the SA chains but also acts as a crosslinking agent, creating a 3D network. This is achieved through the numerous alkyne functionalities on each SA chain and the many azide functionalities on the MXene surface, allowing the SA chain to chemically attach to multiple MX‐QDs and a single MX‐QD to connect with various SA chains. This extensive network formation restricts the movement of SA chains, thus explaining the absence of a *T*_g_. The TGA curves show improved thermal stability in the final polymer nanocomposites with increasing MX‐QD content, enhancing both residual weight and the onset temperature for degradation. This is attributed to the high thermal resistance properties of MX‐QDs^[^ ^37^ ^]^; more details are available in Table S2 (Supporting Information). Based on these findings, all samples exhibit high thermal stability (>200 °C), rendering them ideal for application as a SPE.

### 2.2 Electrochemical Performance

**Figure 2:**
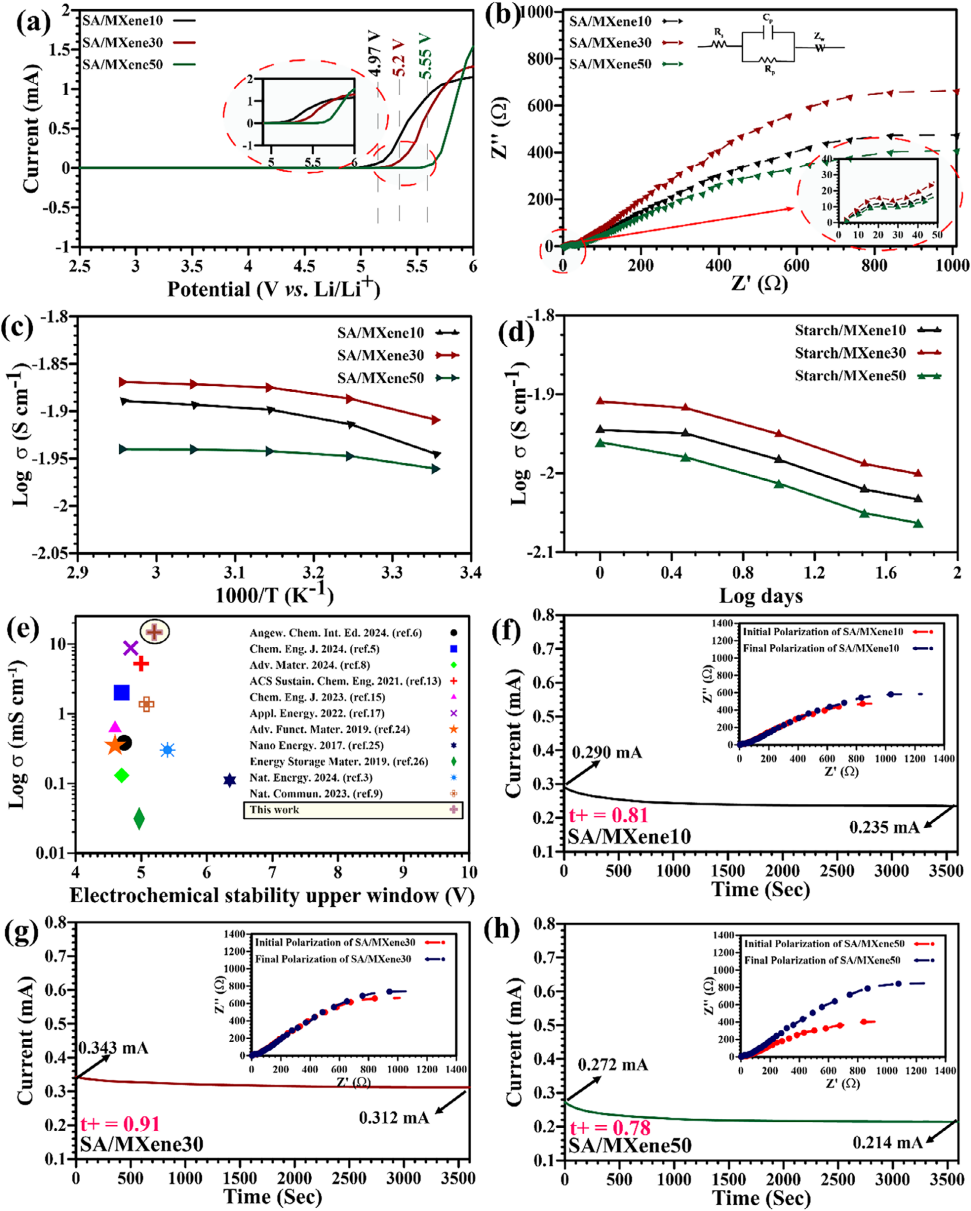
LSV curves (a), Nyquist curves (b), and ionic conductivity versus temperature/days (c and d) for prepared SPEs; (e) comparison of ionic conductivity of divers SPEs; (f to h) chronoamperometry curves demonstrating the time‐dependent response of 10 mV DC polarization for symmetric Li/SPEs/Li cells (the legend figure showcases corresponding AC EIS plots of the cell before polarization and after reaching the steady‐state current).

The results of linear sweep voltammetry (LSV), Nyquist plots, ionic conductivity versus temperature and time, and chronoamperometry for the synthesized SPEs are presented in **Figure** **2**. To assess the potential of SA/MXene nanocomposites in ASSLIBs, LSV was carried out as shown in Figure 2a. While pure SA has an electrochemical stability window up to 4.8 V,^[^ ^17^ ^]^ adding MX‐QDs extends this to 4.97, 5.2, and 5.55 V for 10, 30, and 50 *wt*.% MX‐QDs, respectively. This enhancement in electrochemical stability is attributed to the confined and cross‐linked structure of the SA matrix due to the incorporation of MX‐QDs. The chemically stable 2D structure of MXene sheets provides a robust framework that facilitates this confinement. The confinement effect and cross‐linking restrict the movement of polymer chains, thereby enhancing the electrochemical stability.^[^ ^38^ ^]^ The gradual increase in the stability window also confirms proper MX‐QD dispersion within the SA matrix, as seen in TEM images. This makes these SPEs suitable for high‐voltage ASSLIB applications. On the other hand, the ionic conductivity of SPEs was determined from Nyquist plots using stainless steel blocking electrodes (Figure 2b). The circuit model shown as an inset in Figure 2b provides the electrode/electrolyte interface resistance (*R*_s_) and polymer electrolyte resistance (*R*_p_); these values are used to calculate ionic conductivity (see supporting information for detailed calculations, Equation S1 and Figure S2, Supporting Information). The ionic conductivity (σ) for SA/MXene10 is 11.3 mS cm^−1^, which increases to 14.8 mS cm^−1^ for SA/MXene30 and then decreases to 10.8 mS cm^−1^ in SA/MXene50. Notably, the conductivity of pure SA is 8.76 mS cm^−1[^ ^17^ ^]^ while the ionic conductivity of LiPF_6_ (1 M) in dimethyl carbonate/diethyl methyl carbonate (1:1 v/v), a common liquid electrolyte for LIBs, is 12.5 mS cm^−1^ at room temperature.^[^ ^39^ ^]^ Thus, incorporating 30 *wt*.% MX‐QDs into the SA matrix can produce an SSE with superionic conductivity surpassing that of widely used liquid electrolytes. The reasons for this phenomenon and the drop in σ for the SA/MXene50 electrolyte (despite no MX‐QDs aggregation) will be discussed after examining the behavior of σ versus temperature/time and the cation transfer numbers of the SPEs.

As depicted in Figure 2c, increasing the temperature from 25 to 65 °C increases the ionic conductivity, and this increase is linear on a semi‐log plot of σ versus 1/T (Nyquist plots can be found in Figure S3a–c, Supporting Information). This rise is most pronounced for SA/MXene10 (from 11.3 to 13.9 mS cm^−1^) and least for SA/MXene50 (from 10.8 to 12.7 mS cm^−1^), attributed to the augmentation of both local segmental motion of polymer chains and mobile carrier ions.^[^ ^23^ ^]^ Despite the conductivity rise with temperature (line slope), this increase is not as significant as compared to other SPEs.^[^ ^10^, ^12^, ^19^, ^20^, ^21^, ^22^, ^23^ ^]^ The rationale behind this lies in the highly crosslinked structure of the prepared SPEs. As mentioned, SA itself possesses a high *T*_g_ (≈150 °C), well exceeding the maximum test temperature of 65 °C. Consequently, the increase in temperature has an insignificant effect on the motion of SA chains. Moreover, the addition of MX‐QDs, serving as both solid filler and crosslinker, further restricts chains motion.^[^ ^36^, ^40^ ^]^ All the prepared SPEs exhibit temperature behavior following the Arrhenius model (Equation S3, Supporting Information), facilitating the calculation of activation energy (*E*_a_) and pre‐exponential factor (*A*), as their values are displayed in Table S3 (Supporting Information).^[^ ^41^ ^]^ Notably, our synthesized SPEs exhibit a remarkably lower *E*_a_ compared to other studies using different SPEs (detailed discussions regarding *E*_a_ and *A* are also available in Section S2.2, Supporting Information).

The changes in ionic conductivity over 3, 10, 30, and 60 days after the initial electrochemical impedance spectroscopy (EIS) test for SPEs are depicted in Figure 2c,d, with detailed Nyquist curves provided in Figure S3d–f (Supporting Information). The smallest decrease in σ is observed for SA/MXene30, which is ≈13.4%, followed by 18.6% for SA/MXene10, and the highest decline of 26% for SA/MXene50. Despite this 26% reduction after two months, the ionic conductivity of SA/MXene50 remains significantly better than other polymer electrolytes reported in the literature, particularly those based on synthetic polymers.^[^ ^10^, ^11^, ^12^ ^]^ To provide more rigorous mechanistic insights for the reason behind the high ionic conductivity of the SA/MXene SPEs, we estimated the cation transfer number (*t*+), by both electrochemical impedance spectroscopy as well as potentiostatic/chronopotentiometry polarization studies (Figure 2f–h).^[^ ^23^ ^]^ Employing the Vincent–Evans equation (Equation S2, Supporting Information) allows the estimation of the *t*+ numbers for SA/MXene10, SA/MXene30, and SA/MXene50, which are found to be 0.81, 0.91, and 0.78, respectively (see Table S4, Supporting Information for more information). Typically, *t*+ falls within the range of 0.2 to 0.4 for liquid electrolytes,^[^ ^42^ ^]^ ≈0.5 to 0.6 for synthetic polymer electrolytes,^[^ ^10^, ^23^ ^]^ and 0.7 to 0.8 for natural‐based polymers.^[^ ^14^, ^16^, ^17^ ^]^ A higher *t*+ is advantageous in preventing the growth of the lithium dendrites, mitigating polarization effects, and enhancing the stability of batteries. Conversely, a lower *t*+ diminishes the transport capacity of cations owing to the impediment posed by mobile anions, thereby fostering dendritic lithium deposition and propagation.^[^ ^23^, ^43^ ^]^ The SA/MXene‐based SPEs exhibit low polarization due to starch acetate's oxygen‐rich matrix, which facilitates efficient Li⁺ hopping, and MXene quantum dots’ Ti atoms, which stabilize PF₆⁻ anions to maintain high available Li⁺ concentrations. This results in minimal initial‐to‐steady‐state current differences, as shown in Figure 2f–h. These properties ensure selective Li⁺ conduction, distinguishing SA/MXene from conventional SPEs with higher polarization and lower t⁺ values. Therefore, the achieved *t*+ value of 0.91 is unprecedented in existing studies, holding great promise for achieving stable cyclic performance in the final battery configuration. This increase is in line with the literature, employing electron acceptor fillers within polymer electrolytes to increase the *t*+ number. For instance, Chen et al. demonstrated that incorporating lithium montmorillonite into the poly(ethylene carbonate) (PEC) matrix increased the *t*+ from 0.45 to 0.83.^[^ ^24^ ^]^

**Figure 3:**
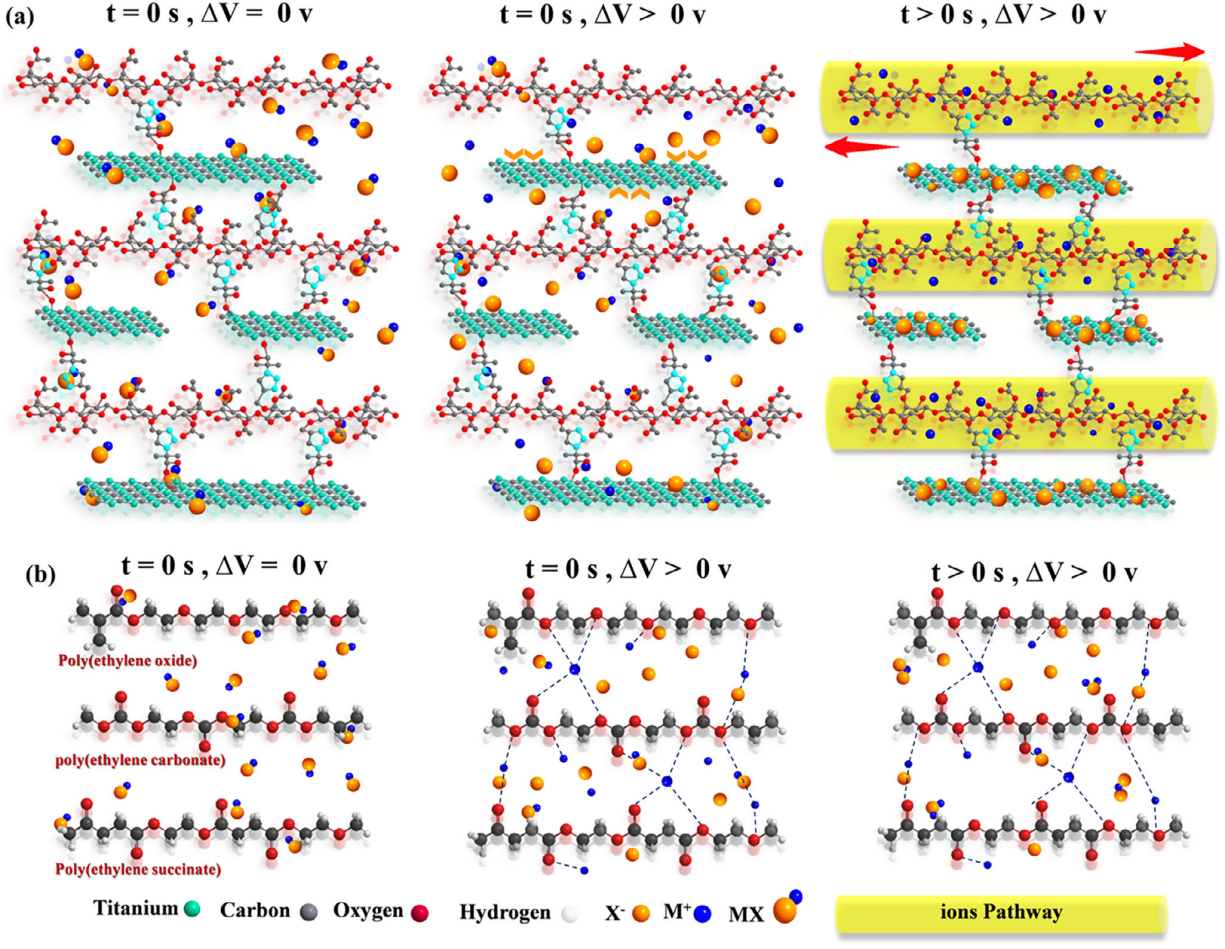
Schematic of Li^+^ stabilization and its movement in the SA/MXene (a) and commercial solid polymer (b) electrolytes in three stages. Blue dashed lines are the coordination between the electron donor atoms and the lithium cation; the abundance of these coordination centers in the SA/MXene electrolyte results in the creation of pathways (yellow cylinders) for easy Li^+^ movement. (This figure is a schematic illustration intended to conceptually represent the proposed Li⁺ ion transport mechanism; it is not derived from any computational modeling.).

However, questions persist regarding the high ionic conductivity of prepared SPEs (see Figure 2e), their elevated cation transfer number, sustained ionic conductivity over two months, low activation energy, and other related aspects. The most reliable sound explanation for these questions lies within the SPEs’ nanostructure. For a thorough understanding, the molecular structure of our devised SPEs based on SA /MX‐QDs (**Figure** **3**a) are compared with those of well‐known synthetic polymer electrolytes, including polyethylene oxide (PEO), poly(ethylene carbonate) (PEC), and poly(ethylene succinate) (PES) (Figure 3b). This comparison is conducted across three distinct stages: first, Δ*V* = 0 and *t* = 0, representing the initial state before applying any potential difference (solely lithium salt (MX) in the electrolyte); second, Δ*V* ≠ 0 and *t* = 0, signifying the moment when a potential difference is applied (initiating the dissociation of MX into M^+^ and X^‐^); and thirdly, Δ*V* ≠ 0 and *t* ≠ 0, representing the performance of the mentioned polymer electrolytes at longer times. In the initial stage, the primary distinctions lie in the composition of atoms and the free volume within the polymer electrolytes. In the developed SPEs of this study, a significant amount of oxygen (O) atoms exists in the polymeric backbone. For instance, in a 65 Å‐length SA chain, there are roughly 86 O atoms, whereas PEO, PEC, and PES contain 25, 33, and 29 O atoms, respectively; furthermore, the surface of the MX‐QDs contains oxygen‐enriched functional groups. This implies that the SA/MXene‐based electrolytes offer more sites for coordination with Li^+^. Additionally, the presence of electron‐acceptor atoms such as Ti in the SA/MXene electrolyte further distinguishes it from other synthesized polymer‐based electrolytes. Therefore, the ability to stabilize X^‐^ ions is unique to the SA/MXene system. The final distinction lies in the available free volume within the polymer electrolytes. In the SA/MXene electrolytes, the presence of large acetate functional groups results in a significant steric hindrance between the polymer chains and with MX‐QDs. This creates ample free volume between the electrolyte consistent, facilitating Li^+^ movement, a characteristic not observed in the dense PEO, PEC, or PES polymers.

Now, moving to the second stage, upon applying the potential difference, MX salt begins to dissociate into M^+^ and X^‐^; some undissociated salts may still be present at this stage. In SA/MXene electrolytes, the abundance of O atoms and the presence of Ti atoms facilitate the instant stabilization of both M^+^ and X^‐^ ions, thereby preventing further irreversible reactions of the diverse charge species. However, in other SPE systems, the limited number of available O atoms stabilizes only a few M^+^ ions. Additionally, the absence of electron acceptor atoms renders all X^‐^ ions unstable in these systems. Consequently, the majority of M^+^ ions would engage in side reactions with X^‐^ ions or undissociated salts (MX) and be excluded from facilitating conductivity. It is noteworthy that many researchers hypothesize that long‐chain polymer motion is vital for ion conduction.^[^ ^11^, ^23^ ^]^ This holds true for polymers with few O atoms in their repeating unit, where motion exposes new coordination sites for M^+^. However, in polymers like starch acetate, rich in O atoms, chain motion has a minimal effect on the ion transfer. Li^+^ ions can freely move through numerous coordination sites due to the applied potential difference, not limited to polymer chain motion.^[^ ^16^, ^17^ ^]^

During the final stage, in the SA/MXene systems, over time, X^‐^ ions gradually migrate toward the surface of the MX‐QDs, owing to the coordination with Ti; the abundance of O atoms in the SA chains creates pathways for the smooth and unhindered movement of M^+^ ions between the two electrodes (see Figure S4, Supporting Information, yellow cylinders paths), ensuring stability for both charged species. However, in the other SPE systems, as depicted in Figure 3b, there are various charged species present alongside M^+^ (including M_2_X^+^ and MX_2_^‐^). The presence of these additional charged species within the SPE results in a low concentration of M^+^ ions, leading to low ionic conductivity. Furthermore, the gradual loss of M^+^ ions, attributed to their participation in the side reactions with undissociated MX or other charged species, combined with the inability of these charged species to engage in reversible reactions on the surface of the electrode, results in a gradual decrease in battery cyclic performance over time.^[^ ^44^ ^]^ The cation transfer number supports this hypothesis, as higher *t*+ values indicate greater Li^+^ ions stability and reduced participation in side reactions. The well‐designed SA/MXene‐QDs electrolyte structure enables effective stabilization of both M^+^ and X^‐^, sustaining ionic conductivity. However, the reduced conductivity in SA/MXene50 is attributed to the lower oxygen atom content (reduced share of SA), resulting in decreased coordination centers for M^+^ and reduced ion pathways, making cation movement and stabilization more difficult.

As discussed, the SA/MXene electrolyte has demonstrated the highest ionic conductivity among other reported SPEs to date. However, it is even higher than the liquid electrolyte case. To explain this, we consider the following. The mobility of ions in a liquid medium is significantly higher than in a solid one; however, the key factor is the number of ions. As mentioned, in the best‐case scenario for liquid electrolytes, *t*+ is equal to 0.4; whereas, for the SA/MXene30, this number is 0.91. Therefore, in the SA/MXene30 electrolyte, there are more than twice as many Li^+^ ions compared to the liquid electrolyte. Thus, this high number of ions (M^+^) can completely compensate for the issue of ion mobility.

**Figure 4:**
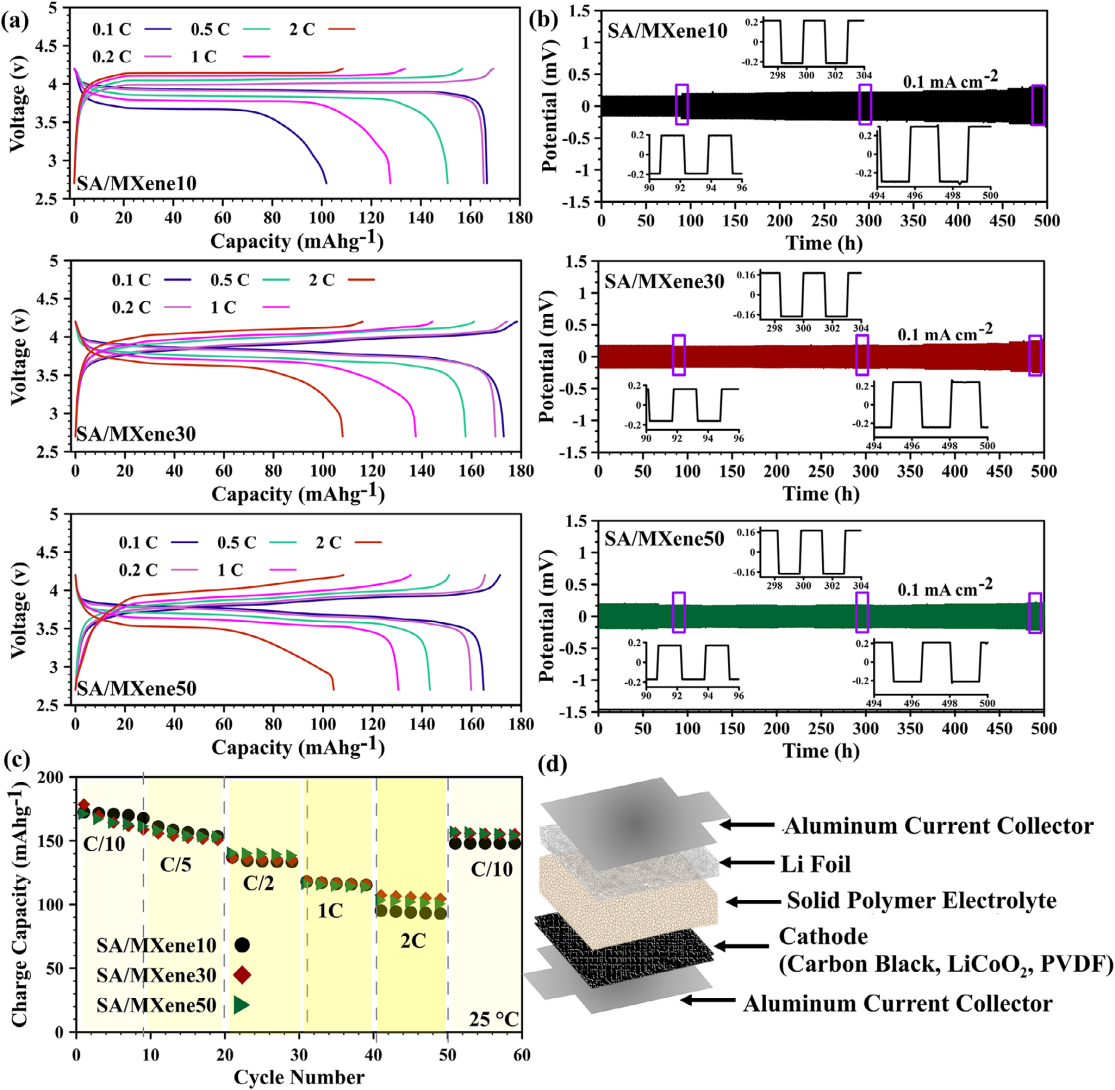
(a) Galvanostatic charge−discharge profiles of LiCoO_2_/SPEs/Li at different current densities (0.1, 0.2, 0.5, 1, and 2 C); (b) the potential profiles and Li/SPEs/Li symmetric battery with current density of 0.1 mA cm^−2^ over 500 h at 25 °C; (c) rate performance of SPEs at various C rates; (d) schematic of the ASSLIBs cell assembly.

**Figure** **4**a shows the typical charge–discharge profiles of the LiCoO_2_/SPEs/Li batteries within a voltage range of 3–5 V at various rates from 0.1 C to 2 C at 25 °C (the structure of the assembled cell can be schematically seen in Figure 4d). The prepared ASSLIBs exhibit charge capacities of 170.31, 174.57, and 165.40 mAh g⁻¹ for SA/MXene10, SA/MXene30, and SA/MXene50, respectively, at a rate of 0.2 C. A comparative analysis with literature reports employing identical LiCoO₂ cathodes reveals that the SPEs developed in this study exhibit exceptional stability against high‐voltage cathodes and lithium anodes. Furthermore, their high ionic conductivity and minimal polarization facilitate efficient Li^+^ transport, enabling optimal cathode utilization. The theoretical capacity of LiCoO₂ cathodes, characterized by their crystalline structure, approaches 160–170 mAh g⁻¹ at a 4 V potential difference.^[^ ^17^ ^]^ However, conventional SPEs often fall short, yielding capacities of ≈140–150 mAh g⁻¹ due to limited ionic conductivity and compromised electrode stability.^[^ ^11^, ^12^, ^13^, ^19^, ^20^, ^21^, ^22^, ^23^ ^]^ In contrast, the SA/MXene SPEs presented herein provide a conducive environment for Li^+^ migration, allowing for the full exploitation of LiCoO₂ cathode capacity during charging.

Elevated currents induce polarization, widening the gap between charge and discharge voltages, leading to Li^+^ consumption, dendrite growth escalation, and, thus, ASSLIBs capacity reduction.^[^ ^45^, ^46^ ^]^ Consequently, capacity decline varies, from 35.32% for the LiCoO_2_/SA/MXene50/Li ASSLIB to 41.76% for the LiCoO_2_/SA/MXene10/Li ASSLIB, as the current increases from 0.1 C to 2 C. As expected, a higher proportion of solid MX‐QDs improves the mechanical strength of the SPE, thus mitigating dendrite growth (and lowering capacity reduction with increased current). However, further analysis is needed to validate this hypothesis. Furthermore, the relatively low reduction in the battery capacity based on SA/MXene electrolyte (≈40% when the current density increases from 0.1 to 2C) can be attributed to the high number of electron donor atoms in the electrolyte, which provide numerous coordination sites for Li⁺ ions. Consequently, at higher currents, these abundant coordination sites prevent the limitations in cation movement. Typically, most highly ionic conductive solid polymer electrolytes (except for those developed in this study) exhibit a 40% decrease in charge capacity when the current increases from 0.1 to 1C.^[^ ^13^, ^14^, ^15^, ^24^, ^25^, ^26^ ^]^

The voltage gap between charge and discharge curves represents the total cell overpotential, comprising twice the sum of the Ohmic drop, kinetic, and mass transfer overpotentials for both cathode and anode. The relatively small voltage gap observed indicates minimal kinetic and mass transfer losses, as well as low Ohmic resistance, highlighting the high conductivity and efficient ion transport in SA/MXene‐QDs electrolytes. However, the non‐zero voltage gap suggests some limitation in ion movement and penetration into the electrode, which is inherent to solid‐state electrolytes compared to their liquid counterparts.^[^ ^17^, ^47^ ^]^

The stability and interfacial contact between the Li metal anode and SPEs were assessed for 500 h through polarization analysis of a symmetric battery under a current density of 0.1 mA cm⁻^2^ (Figure 4b). All cells exhibited excellent plating/stripping performance, with low initial overpotentials of 15, 17, and 19 mV for SA/MXene10, SA/MXene30, and SA/MXene50, respectively. These correspond to initial ionic conductivities of 2.78, 2.45, and 2.19 mS cm⁻¹ (calculated using the resistance, R = ΔV/J; where ΔV is the overpotential during Li plating/stripping and J is the applied current density). These values are lower than those from EIS measurements (11.3, 14.8, and 10.8 mS cm⁻¹ for SA/MXene10, SA/MXene30, and SA/MXene50, respectively), and the trend (SA/MXene10 > SA/MXene30 > SA/MXene50) differs from EIS (SA/MXene30 > SA/MXene10 > SA/MXene50). The lower conductivity in plating/stripping tests compared to EIS is attributed to the inclusion of interfacial resistance at the Li/SPE interfaces, which is not present in EIS measurements using stainless steel blocking electrodes. In plating/stripping tests, the resistance encompasses both the bulk SPE resistance and the Li/SPE interfacial resistance, which can be significant due to dynamic cycling and potential formation of interfacial layers.^[^ ^5^ ^]^ The MXene‐QDs play a critical role in mitigating this interfacial resistance by stabilizing PF₆⁻ anions via Ti coordination, reducing ion pairing, and promoting uniform Li⁺ deposition over time. This enhances the compatibility of the SPE with the lithium metal anode, contributing to the low initial overpotentials observed.

It can be observed, at the beginning of the test, SA/MXene10 has the highest ionic conductivity due to the high number of oxygen atoms in the SA matrix, which interact with Li electrodes and reduce interfacial resistance. However, in long‐term performance, MXene‐QDs are beneficial for the formation of a stable, dendrite‐resistant interface with the lithium anode, leading to lower interfacial resistance. Consequently, at the end of the plating/stripping test, SA/MXene50 shows the highest ionic conductivity (the same trend can be seen in cyclic performance). After 500 h, the final overpotentials were 29, 23.5, and 20 mV for SA/MXene10, SA/MXene30, and SA/MXene50, corresponding to ionic conductivities of 1.44, 1.77, and 2.08 mS cm⁻¹, respectively. This reversal in trend highlights the long‐term benefits of higher MXene‐QDs content in stabilizing the Li/SPE interface. Remarkably, all SPEs demonstrated stable cycling for over 500 h without short‐circuiting, a significant improvement over liquid electrolytes, which typically fail before 200 h due to dendrite growth,^[^ ^3^, ^40^ ^]^ and PEO‐based SPEs, which short‐circuit within 60–300 h.^[^ ^10^ ^]^ The stable long‐term performance is driven by the SPEs’ dendrite growth resistance, stemming from the enhanced mechanical properties imparted by MXene‐QDs acting as high‐modulus fillers. The uniform dispersion of MX‐QDs within the SA matrix prevents dendrite penetration, while their anion‐stabilizing properties minimize interfacial degradation over time. Additionally, the low initial and final overpotentials are associated with the high ionic conductivity facilitated by the oxygen‐rich SA matrix, which enables rapid and uniform Li⁺ diffusion, and the MXene‐QDs, which enhance Li⁺ availability at the interface. The exceptional stability of SA/MXene50, with a minimal overpotential increase (1 mV), underscores the role of MXene‐QDs in creating a mechanically robust and electrochemically stable structure. This is further supported by the minimal capacity fade in SA/MXene50‐based all‐solid‐state lithium‐ion batteries at increasing current densities, highlighting its potential for practical applications.

Figure 4c illustrates the rate performance of the ASSLIBs (LiCoO_2_/SPEs/Li) at different current densities. Generally, an increase in C rates typically results in a decrease in battery capacity across all ASSLIBs. However, a notable decline in capacity is observed in the first 20 cycles for all ASSLIBs, occurring at relatively similar current densities (0.1 and 0.2 C); subsequently, the capacity stabilizes for each C rate. This initial decrease may be attributed to dendrite growth in the early cycles, with the SPEs potentially mitigating further growth.^[^ ^6^, ^48^ ^]^ However, further investigation into the long‐term cyclic charge–discharge performance of ASSLIBs is needed to confirm this hypothesis. Overall, the rate performance of the ASSLIBs demonstrates favorable and consistent cyclic performance, attributed to the high ionic conductivity of the SPEs and their effective interaction with the electrodes. Particularly noteworthy is the SA/MXene50 electrolyte, which exhibits an initial capacity lower than SA/MXene10. However, after 60 cycles, it surpasses SA/MXene10 in capacity, owing to its superior mechanical properties.

**Figure 5:**
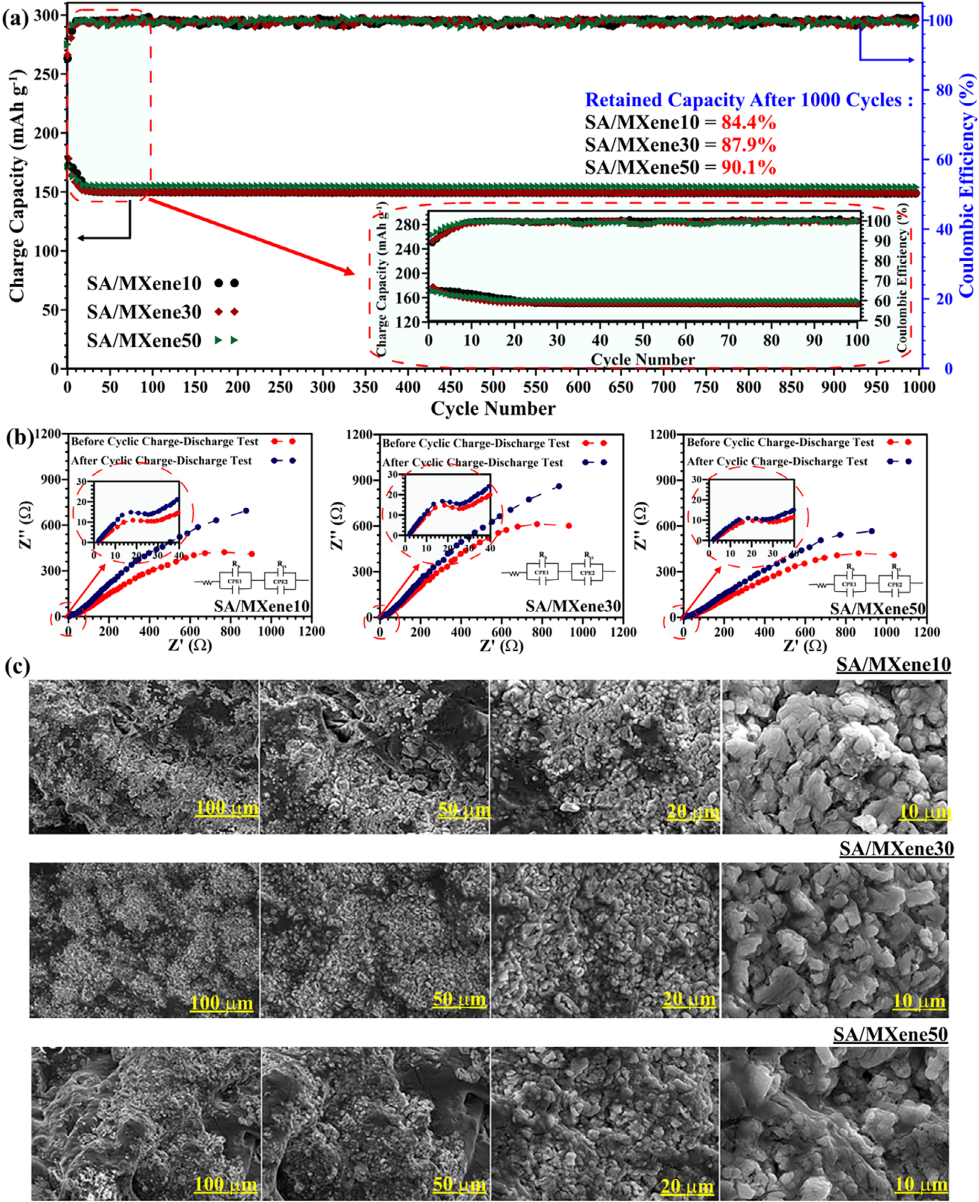
(a) Charge capacities and Coulombic efficiency at 0.1 C rate of the LiCoO_2_ /SPEs/Li for 1000 cycles; (b) Half‐cell EIS plots of SA/MXene10, SA/MXene30, and SA/MXene50 before and after cyclic charge–discharge test. (c) FE‐SEM results of SPEs after 1000 charge–discharge cycles.

The enduring cyclic performance of the ASSLIBs at 0.1C over 1000 cycles, which is a crucial electrochemical analysis, alongside their coulombic efficiency (CE), is illustrated in **Figure** **5**a (see the detailed data in Table S5, Supporting Information). All the prepared ASSLIBs exhibit exceptional cyclic stability over 1000 charge–discharge cycles. This stability is attributed to the numerous stable pathways for Li⁺ ions facilitated by the oxygen‐rich SA polymer chains. Additionally, MX‐QDs serve to stabilize the X⁻ ions, preventing their reaction with Li⁺ and, thus, preventing their removal from the charge–discharge cycles. Moreover, MX‐QDs play a critical role in regulating the solid electrolyte interphase (SEI) and cathode electrolyte interphase (CEI), ensuring long‐term cycling stability. The SEI formation at the Li metal anode is crucial for battery performance, as an unstable SEI layer leads to continuous electrolyte decomposition, increased interfacial resistance, and dendrite growth.^[^ ^13^, ^24^ ^]^ MX‐QDs contribute to SEI stabilization by forming an inorganic‐rich SEI layer composed of LiF and Li₂CO₃, which enhances ionic conductivity and suppresses lithium dendrites.^[^ ^49^ ^]^ Furthermore, the high surface area and functional groups of MX‐QDs promote uniform lithium‐ion distribution and facilitate homogeneous lithium deposition, thereby mitigating SEI instability.^[^ ^50^ ^]^ On the cathode side, the CEI formation is equally critical, as CEI degradation can lead to capacity fading in high‐voltage cathodes such as LiCoO₂. The CEI layer, primarily composed of LiF, Li₂CO₃, and metal oxides, forms due to electrolyte oxidation at high voltages. In SPE‐based systems, CEI stability is often improved due to better electrode‐electrolyte contact compared to inorganic solid electrolytes.^[^ ^51^ ^]^ MX‐QDs further contribute to CEI stabilization by preventing excessive electrolyte decomposition and minimizing the formation of resistive CEI layers, which is a common issue in high‐voltage LIBs.^[^ ^15^, ^17^, ^52^ ^]^

The retained capacity (RC) after 1000 charge‐discharge cycles for LiCoO₂/SA/MXene10/Li, LiCoO₂/SA/MXene30/Li, and LiCoO₂/SA/MXene50/Li was 84.4, 87.9, and 90.1%, respectively, serving as additional evidence of the extraordinary mechanical resistance of SA/MXene50 against dendrite growth. In previous studies, most SPEs demonstrate capacity retention of ≈90% only for 100–1000 cycles, particularly when natural polymers are used as electrolytes.^[^ ^15^, ^16^, ^17^ ^]^ The CE, representing the discharge capacity over the charge capacity for each cycle, is ≈90% for all ASSLIBs at the first cycle and rapidly increases to over 99%, emphasizing the effective role of MXene‐stabilized SEI/CEI layers in facilitating lithium‐ion transport while minimizing side reactions.^[^ ^25^, ^49^, ^50^, ^52^ ^]^ The EIS results from the half‐cell tests indicate a reduction in electrolyte resistance following charge‐discharge cycling, as shown in Figure 5b. This can be attributed to enhanced electrode/electrolyte compatibility during the initial cycles and the formation of a stabilized SEI/CEI layer, which helps lower charge transfer resistance at both interfaces.^[^ ^13^, ^14^, ^49^ ^]^

The FE‐SEM images of the Li metal anode after 1000 cycles provide direct evidence of the dendrite‐suppressing effect of MX‐QDs in the SA‐based SPEs. As shown in Figure 5c, all samples exhibit a homogeneous lithium deposition pattern, but a clear trend of reduced dendrite size and uniform lithium plating is observed as the MX‐QD content increases. For LiCoO₂/SA/MXene10/Li, the anode surface exhibits noticeable but controlled dendrite structures, indicating that the polymer electrolyte provides some stabilization but is still prone to lithium microstructures forming over extended cycling. In LiCoO₂/SA/MXene30/Li, the dendrite structures appear finer and more evenly distributed, suggesting improved SEI formation that regulates lithium‐ion transport. The LiCoO₂/SA/MXene50/Li sample, however, shows a remarkably smooth surface with almost no visible lithium protrusions, highlighting the superior dendrite resistance conferred by MX‐QDs’ high mechanical strength and ion‐channeling effects.^[^ ^12^, ^49^, ^50^, ^51^, ^52^ ^]^ This behavior is attributed to three main factors: 1) MX‐QDs facilitate a more LiF‐rich SEI composition, as LiF is known to promote a uniform Li‐ion flux at the anode/electrolyte interface^[^ ^13^, ^24^ ^]^; 2) the crosslinking effect of MX‐QDs within the SA matrix reinforces the mechanical properties of the SPE, preventing lithium metal penetration and crack formation^[^ ^12^ ^]^; the high surface area and polar functional groups of MX‐QDs enhance Li⁺ nucleation homogeneity, preventing local concentration gradients that trigger uncontrolled dendrite growth.^[^ ^13^, ^24^, ^49^, ^51^ ^]^ Furthermore, the absence of large dendritic structures in SA/MXene50‐based cells suggests that dendrite growth is halted after the initial few cycles, as also supported by the stable capacity retention beyond 1000 cycles. This confirms that the optimized electrolyte formulation not only suppresses dendrite growth but also stabilizes the SEI composition throughout prolonged cycling, aligning well with observations from the literature.^[^ ^49^ ^]^ The results confirm that SEI and CEI stabilization through MX‐QDs plays a vital role in improving cycle life and interfacial stability in ASSLIBs. The combination of LiF‐rich SEI formation, enhanced Li⁺ transport, and minimized CEI degradation at the cathode contributes to the excellent electrochemical performance of SA/MXene‐based SPEs. The FE‐SEM analysis further reinforces that the incorporation of MX‐QDs effectively suppresses dendrite growth and promotes a stable lithium deposition morphology, further solidifying the potential of SA/MXene SPEs for long‐term lithium‐metal battery applications.

The SPEs designed in this study by grafting SA to MX‐QDs via click reaction exhibit outstanding electrochemical performance, characterized by high ionic conductivity, high cation transference number, wide electrochemical stability window, high final battery capacity, and excellent dendrite growth resistance. These safe and environmentally friendly SPEs have the potential to revolutionize liquid electrolyte‐based LIBs, even on an industrial scale, and represent a significant step toward real, sustainable energy storage systems. Specifically, SA/MXene30 is suitable for applications requiring high ionic conductivity and capacity, while SA/MXene50 is ideal for applications demanding long‐term performance.

## 3 Conclusion

Starch acetate, a natural polymer with a unique structure featuring a high oxygen atom density, exhibits good ionic conductivity. To further enhance its electrochemical properties, MX‐QDs filler was introduced, leveraging its electron acceptor atoms to stabilize the negative part of the dissociated lithium salt (X^‐^) and its high mechanical properties to increase the dendrite growth resistance. CuAAC click chemistry enabled uniform dispersion of MX‐QDs up to 50% within the starch acetate matrix. The resulting SA/MXene50 nanocomposite demonstrated outstanding electrochemical performance: 14.8 mS cm^−1^ ionic conductivity, 0.91 cation transfer number, 5.2 V electrochemical stability window, 175 mAh g^−1^ battery capacity, and 88% retained capacity after 1000 cycles. The unique structure of the synthesized SPE balances electron donor and acceptor atoms, stabilizing Li^+^ and M^‐^ ions, and facilitating Li^+^ movement. The SPE's mechanical properties and electrode compatibility further enhance its performance, making it an ideal candidate for ASSLIBs. This work presents a safe, inexpensive, eco‐friendly, and efficient alternative to liquid electrolytes, paving the way for sustainable energy storage systems based on ASSLIBs. The developed SPEs have the potential to revolutionize the energy storage landscape, enabling widespread adoption of renewable energy sources.

## 4 Experimental Section

The chemical grafting of SA chains (Sections S1.1 and S1.2, Supporting Information) onto the MXene surface (Sections S1.1 and S1.3 and Figure S1a, Supporting Information) was carried out using a CuAAC click reaction. Typically, this click reaction occurs between alkyne (─C≡CH) and azide (N_3_) functionalities^[^ ^53^ ^],^ which were not inherently present in MX‐QDs or SA. Therefore, in a two‐step modification process, N_3_ functionalities were first introduced onto the surface of the MXene, followed by the addition of alkyne groups onto the SA chains in a one‐step method.

### Modification of Ti_3_C_2_T_x_ MXene Nanosheets

To obtain MXene with azide functionality (MXene‐N_3_), a mixture of Ti_3_C_2_T_x_ MXene nanosheets (0.2 g) and α‐bromoisobutyryl bromide (BiBB) (5 mL) was dissolved in 25 mL *N*,*N*‐dimethylformamide (DMF) and stirred for 24 h at 60 °C. After completion of the reaction, the mixture was washed with methanol to yield MXene nanosheets with bromine groups (MXene‐Br). Subsequently, MXene‐Br was dried for 24 h at 60 °C in a vacuum oven (with a reaction efficiency of 90%, determined gravimetrically). Next, to obtain MXene‐N_3_, 0.1 g MXene‐Br was dissolved in 10 mL DMF, and 0.2 g sodium azide (NaN_3_) in 15 mL DMF was added dropwise over 15 min. The reaction mixture was stirred for 48 h at 60 °C, washed with 30 mL methanol, and centrifuged (Figure 1a). Finally, MXene‐N_3_ nanosheets were dried in a vacuum oven at 65 °C for 24 h (with a reaction yield of 88% based on weight). Furthermore, during these modification steps, the use of probe and bath sonication for mixing results in the reduction of MXene nanosheets' size, yielding the final product MXene quantum dots (MX‐QDs).^[^ ^54^ ^]^ The transmission electron microscopy (TEM) images of the MXene‐Br and MXene‐N_3_ can be found in Figure S1b,c (Supporting Information), respectively. FT‐IR spectra, XRD patterns, and TGA results are presented in Figures S1d–f (Supporting Information), respectively. Also, XPS results are presented in Figure 1b–f.

### Modification of Starch Acetate by Propargyl Bromide

1.0 g amorphous starch acetate (with a degree of substitution equal to 3) and 0.2 g potassium hydroxide (KOH) were dissolved in 23 mL distilled water over a period of 6 h at 25 °C. Subsequently, 5 mL of propargyl bromide dissolved in 13 mL of toluene was added to the reaction mixture, which was stirred for 72 h at 60 °C under a nitrogen atmosphere (see Figure 1g). The resulting precipitate was purified by filtration using filter paper, yielding a white‐creamy solid. The obtained product, alkyne‐modified SA, was then dried for 24 h at 60 °C in a vacuum oven. The reaction yield was determined 80% using a gravimetric method. Modification was approved using FT‐IR and NMR spectroscopies, respectively (Figure 1h,i).

### Chemical Grafting and SPEs Preparation

Through the process shown in Figure 1a,j, alkyne SA was grafted onto the MX‐QDs (SA/MXene) synthesized via a click reaction process (Figure 1j). Initially, specific quantities of MXene‐N₃ were dispersed in 10 mL dimethyl sulfoxide (DMSO) using a probe sonicator, and subsequently, the mixture was added to alkyne SA. For SA/MXene10, SA/MXene30, and SA/MXene50, the respective amounts of MXene‐N₃ were 0.02, 0.06, and 0.1 g, while the corresponding quantities of alkyne modified SA were 0.18, 0.14, and 0.1 g, ensuring consistent ratios in the reaction mixture to optimize grafting efficiency (sample symbols and their integrals can be seen in Table S1, Supporting Information). Subsequently, 0.001 g copper salt (copper bromide) was introduced as a catalyst. The reaction was carried out at 60 °C for 72 h. Then, to purify the catalyst, the mixture was washed with methanol several times. Thermogravimetric analysis (TGA) curves (Figure S1, Supporting Information) exhibit no weight loss peaks associated with DMSO evaporation (expected below 200 °C) or degradation of other small molecular residuals (such as copper bromide), confirming a pure SA/MXene matrix. Finally, a solution of lithium hexafluorophosphate (LiPF₆) as lithium salt (with a ratio of 16:1 units of electron donor atoms per unit of LiPF₆) in DMSO was prepared. The 16:1 ratio was carefully selected to optimize the electrochemical properties of the resulting SPEs. Lower ratios (excess LiPF₆) lead to ion aggregation and reduced conductivity, while higher ratios (insufficient LiPF₆) limit available charge carriers.^[^ ^16^, ^17^ ^]^ Then, the pre‐dried SA/MXene composites were immersed in the LiPF₆/DMSO solution for 2 h, during which DMSO swelled the polymer matrix, facilitating the uniform diffusion of LiPF₆ throughout the composite. This solution‐based swelling approach was specifically designed to ensure a homogeneous distribution of the lithium salt within the SA/MXene10, SA/MXene30, and SA/MXene50 composites. Subsequently, the resulting slurries were vacuum‐dried at 40 °C for 3 days to ensure the complete removal of DMSO. Finally, the dried samples were then subjected to further electrochemical characterization.

## Conflict of Interest

The authors declare no conflict of interest.

## Author Contributions

S.H. performed in methodology, formal analysis, investigation, writing – original draft, and visualization. M.H. and H.K. performed in formal analysis, investigation, and visualization. M.G. performed in validation, resources. Z.W. performed in validation, resources, writing–review, and editing. M.S.‐K. performed in conceptualization, validation, resources, data curation, writing–review and editing, supervision, project administration, funding acquisition.

## Supporting information



Supporting Information

## Data Availability

The data that support the findings of this study are available from the corresponding author upon reasonable request.
