# Supplementary material for: Starch Acetate Grafted to MXene Composite Surpasses Room Temperature Liquid Electrolyte Performance for All‐Solid‐State Lithium‐Ion Batteries
Source: Adv Sci (Weinh). 2025 Jun 19;12(29):e03285. doi: 10.1002/advs.202503285 (PMC12362791; doi:10.1002/advs.202503285)
Supplement: Supplementary file 1 — Supporting Information [file ADVS-12-e03285-s001.docx]

Supporting Information

Starch Acetate grafted to MXene Composite Surpasses Room Temperature Liquid Electrolyte Performance for All-Solid-State Lithium-Ion Batteries

Saeed Hadad^A,B^, Mahtab Hamrahjoo^A,B^, Homayun Khezraqa^A,B^, Marzieh Golshan^A,B^, Zhaohui Wang^C^, Mehdi Salami-Kalajahi^A,B*^

A. Faculty of Polymer Engineering, Sahand University of Technology, P.O. Box 51335-1996, Tabriz, Iran

B. Institute of Polymeric Materials, Sahand University of Technology, P.O. Box 51335-1996, Tabriz, Iran

C. College of Materials Science and Engineering, Hunan University, Changsha 410082, China

* Correspondence concerning this article should be addressed to:

Mehdi Salami-Kalajahi: Email: m.salami@sut.ac.ir, m.salamikalajahi@gmail.com, Tel. /Fax: +98 41 33459097

# **S1. Experimental section**

## **S1.1. Materials**

Corn starch (Sigma-Aldrich) was dried at 50 ℃ for 24 h before processing. Ti_3_AlC_2_ powders with a particle size of <38 μm (Sigma-Aldrich), lithium fluoride (LiF, Sigma-Aldrich), 12 M hydrochloric acid (HCl, Sigma-Aldrich), isopropyl alcohol (Sigma-Aldrich, 99.8%), methanol (Sigma-Aldrich, 99.8%), ethanol (Merck, 99.8%), sodium hydroxide (NaOH, Sigma-Aldrich, 98%), sodium monochloroacetate (SMCA, Merck, 97%), glacial acetic acid (Sigma-Aldrich, 99%), acetic anhydride (Sigma-Aldrich, 98%), iodine (Sigma-Aldrich, 99.99%), sodium thiosulfate (Merck, 99.99%), acetone (Merck, 99.9%), sodium azide (NaN_3_, Aldrich, 99%), methanol (Merck, 99.9%), dimethyl sulfoxide (DMSO, Merck, 99%), toluene (Merck, 99%), *N*,*N*-dimethylformamide (DMF, DaeJung, 99.5%), potassium hydroxide (KOH, Aldrich, 99%), α-bromoisobutyryl bromide (BiBB, Aldrich, 99%), propargyl bromide (Aldrich, 99%), sulfuric acid (H_2_SO_4_, Merck, 98%), lithium hexafluorophosphate (LiPF_6_, Sigma-Aldrich, 99.99%), lithium cobalt(III) oxide (LiCoO_2,_ Sigma-Aldrich, 99.8%), poly(vinylidene fluoride) (PVDF, Sigma-Aldrich), *N*-methyl-2-pyrrolidone (NMP, Sigma-Aldrich, 99.5%), carbon black (Sigma-Aldrich, 99.95%), and lithium (Sigma-Aldrich) were used as received.

## **S1.2. Preparation of starch acetate (SA)**

Starch acetate (SA) with a degree of substitution of 3, was prepared by a previously reported method (**Scheme S1**) [S1]. Corn starch was mixed with acetic acid, sulphuric acid, and acetic anhydride, then reacted with iodine in a microwave oven at 100 ℃ for 1 h. The mixture was washed with ethanol and water, and the resulting slurry was dried in a vacuum oven at 50 ℃ overnight. The reaction was monitored by adding sodium thiosulfate, which indicated the transformation of iodine to iodide by a colour change from dark brown to colourless.


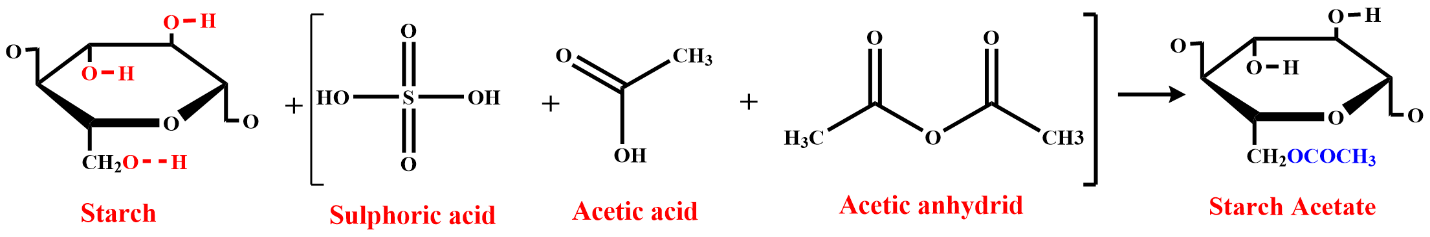


**Scheme S1.** Synthesis mechanism of SA from corn starch

## **S1.3. Preparation of Ti_3_C_2_T_x_ MXene nanosheets**

MXene nanosheets were synthesized by a previously reported method [S2]; *via* selective etching of the aluminum layer from the Ti_3_AlC_2_ MAX phase, using a minimally intensive layer delamination method. LiF was dissolved in HCl and stirred with Ti_3_AlC_2_ for 24 h at 37 °C. The resulting acidic product was washed with water, centrifuged, and sonicated under argon flow to facilitate delamination. The Ti_3_C_2_T_x_ suspension was centrifuged, and the delaminated MXene flakes were collected and stored. The concentration of the MXene solution was determined through gravimetric analysis after vacuum-assisted filtration.

## **S1.4. Characterizations**

Fourier transform infrared spectroscopy (FTIR) spectroscopy was conducted using a Bruker Tensor 27 FT-IR spectrophotometer, spanning 500 to 4000 cm^−1^ with a resolution of 4 cm^−1^. ^13^C NMR (500 MHz) spectra were recorded on a Varian Unity Inova 500 MHz, employing D_2_O as the solvent and tetramethylsilane (TMS) as the internal standard. The morphology of the solid polymer electrolytes’ dendrites were examined using a MIRA3 TESCAN FE-SEM, with samples coated in gold prior to characterization. X-ray diffraction (XRD) analysis was performed on a Siemens D5000 X-ray diffraction instrument with a Cu target (λ = 0.1540 nm) at room temperature. The system, featuring a rotating anode generator operating at 35 kV and 20 mA, scanned samples from 2θ = 5° to 35° in step scan mode. Thermal gravimetric analysis (TGA) was conducted using a PL thermo-gravimetric analyzer (Polymer Laboratories, TGA 1000, UK). Approximately 10 mg of each sample was heated from ambient temperature to 800 °C at a rate of 10 °C min^-1^ under a nitrogen atmosphere at a flow rate of 50 mL min^-1^. Differential scanning calorimetry (DSC) was performed using a NETZSCH DSC 200 F3 instrument (Netzsch Co., Bavaria, Germany), with samples (~10 mg) heated from -40 to 300 °C at a rate of 10 °C min^-1^ under a nitrogen atmosphere at a flow rate of 50 mL min^-1^, after erasing the sample's thermal history. Transmission electron microscope (TEM), Philips CM10, a Zeiss LSM 510 confocal microscope, has been used to investigate the dispersion of the MXene quantum dots (MX-QDs) inside the SA matrix. The elemental compositions of the prepared MXenes, both before and after modification, were analyzed using X-ray photoelectron spectroscopy (XPS) with a Thermo-VG Scientific ESCA Lab 250 microprobe. The instrument was equipped with a monochromated Al K-alpha X-ray source, operated at 200 W, and capable of using monochromatic Al Kα or twin anodes (Mg or Al) as X-ray sources.

## **S1.5. Electrochemical characterization**

The cathode material was prepared by mixing 80 *wt*.% LiCoO₂ (LCO), 15 *wt*.% super-P carbon black, and 5 *wt*.% poly(vinylidene fluoride) (PVDF) as a binder. These components were dispersed in *N*-methyl-2-pyrrolidone (NMP) to create a uniform slurry, which was subsequently coated onto aluminum foil using the doctor blade technique. The coated foil was then dried in a vacuum oven at 80 °C for 24 h to remove residual solvent. The resulting LiCoO₂ cathode had an areal loading of ~ 1.5 mg cm⁻². For cell assembly, solid polymer electrolytes (SPEs) with a disk-shaped area of 36 mm² and a thickness of 1.5 mm were sandwiched between the LiCoO₂ cathode and a lithium foil anode (configured as a thin disc). No adhesives or interlayers were employed in this configuration. The SPE was incorporated as a solid membrane, and its mass and volume per cell correspond directly to the specified dimensions (area: 36 mm², thickness: 1.5 mm). Cell fabrication was performed in an argon-filled glove box to maintain an inert environment for subsequent electrochemical testing. The solid-state cells were assembled using a hydraulic crimper, with an external pressure of approximately 2000 psi (∼13.8 MPa) applied during sealing to ensure robust interfacial contact among the SPE, LiCoO₂ cathode, and lithium metal anode. This pressure was critical to minimizing interfacial resistance and enhancing electrochemical stability. The electronic conductivity of the prepared polymer nanocomposites was measured using a 4-point probe (CDE-4PP) model ResMap178. To measure the ionic conductivity of SPEs, electrochemical impedance spectroscopy (EIS) was conducted using a PGE-18 electrochemical workstation over a temperature range of 25-65 °C, with a frequency range from 1 MHz to 0.1 Hz and an AC potential amplitude of 10 mV. The ionic conductivity of the electrolytes was calculated using the following equation [S3]:

σ = $\frac{h}{R_{p}A}$ (S1)

In the equation, σ represents the ionic conductivity, h stands for the thickness of the polymer film, A denotes the area of the electrode-electrolyte interface, and R_p_ represents the bulk resistance. SPEs were placed between two stainless steel (SS) electrodes for impedance measurements. For the day-dependent EIS tests, the samples were kept at room temperature (not stored in a glovebox) and the same cells were used for measurements without any changes. Cyclic voltammetry (CV) curves were obtained from 0.0 to 6.0 V for the LiCoO_2_/SPE/Li cell using an Atomlab electrochemical workstation at a scan rate of 0.1 mV s^-1^. Galvanostatic charge and discharge (constant current) cycling tests were conducted by the same workstation at room temperature on Li/SPE/LiCoO_2_ cells within the potential window of 2.7 to 4.2 V at various rates. These tests were complemented by EIS measurements taken before and after the polarization scans over a frequency range of 0.1–106 Hz with a 10 mV amplitude at 25 °C.

The transference number (t^+^) of SPEs was determined through DC polarization and impedance analysis, where a 10 mV DC voltage was applied to the Li/SPEs/Li cells. The t^+^ of electrolytes was calculated using the following equation [S4]:

*_Li+_* = $\frac{I_{ss} (\Delta V-I_{0} R_{s.0})}{I_{0}(\Delta V-I_{ss} R_{s,ss})}$ (S2)

In the equation, *I_0_* represents the initial current, *I_ss_* denotes the steady-state currents, *R_s,0_* stands for the initial resistance, and *R_s,ss_* represents the final resistance; assuming a uniform electrode thickness, negligible concentration gradients, and a single dominant ionic species. These assumptions are commonly accepted for simplified systems, but may not hold true for more complex electrolytes. All electrochemical measurements, except for temperature-dependent conductivity, were conducted at room temperature.

# **S2. Results**

## **S2.1 Characterizations**

**Table S1.** Prepared polymer nanocomposites’ symbols and their integrates.

| Sample symbol | Sample integrates |
| --- | --- |
| MXene-Br | MXene sheets modified with bromide functionality |
| MXene-N3 | MXene sheets modified with azide functionality |
| SA/MXene10 | 90 *wt.* % starch acetate and 10 *wt.* % MXene quantum dots |
| SA/MXene30 | 70 *wt.* % starch acetate and 30 *wt.* % MXene quantum dots |
| SA/MXene50 | 50 *wt.* % starch acetate and 50 *wt.* % MXene quantum dots |

The FTIR spectra of MXene nanosheets (**Figure S1d**) exhibit absorbance peaks at approximately 3400, 2900, 1760, 1117, and 608 cm^-1^, corresponding to the stretching vibrations of the carboxyl group (OH), asymmetric and symmetric stretching of C-H bonds, carbonyl (C=O) stretching, C-F stretching, and Ti-O linkage, respectively [S5]. The FTIR spectra of SA (**Figure 1h**) show absorption peaks at 1157 and 1025 (C-O stretching), 3050 to 3650 (O-H bond), and 2960 cm⁻¹ (C-H vibration), along with peaks at 1373 and 1240 cm⁻¹ (CH₃), which are characteristic of the starch acetate polymer backbone [S1].

The ^13^C NMR spectra of SA show signals as follows: C_1_, corresponding to acetyl methyl carbon (CH₃), appears at 20-22 ppm; C_2_, corresponding to acetyl (CH₂), appears at 31-33 ppm; C_3_ and C_4_, corresponding to carbon rings, appear at 58-78 ppm; C_5_ and C_6_, corresponding to anomeric carbon, appear at 96-107 ppm; and C_7_, corresponding to carbonyl carbon (C=O), appears at 178-180 ppm.


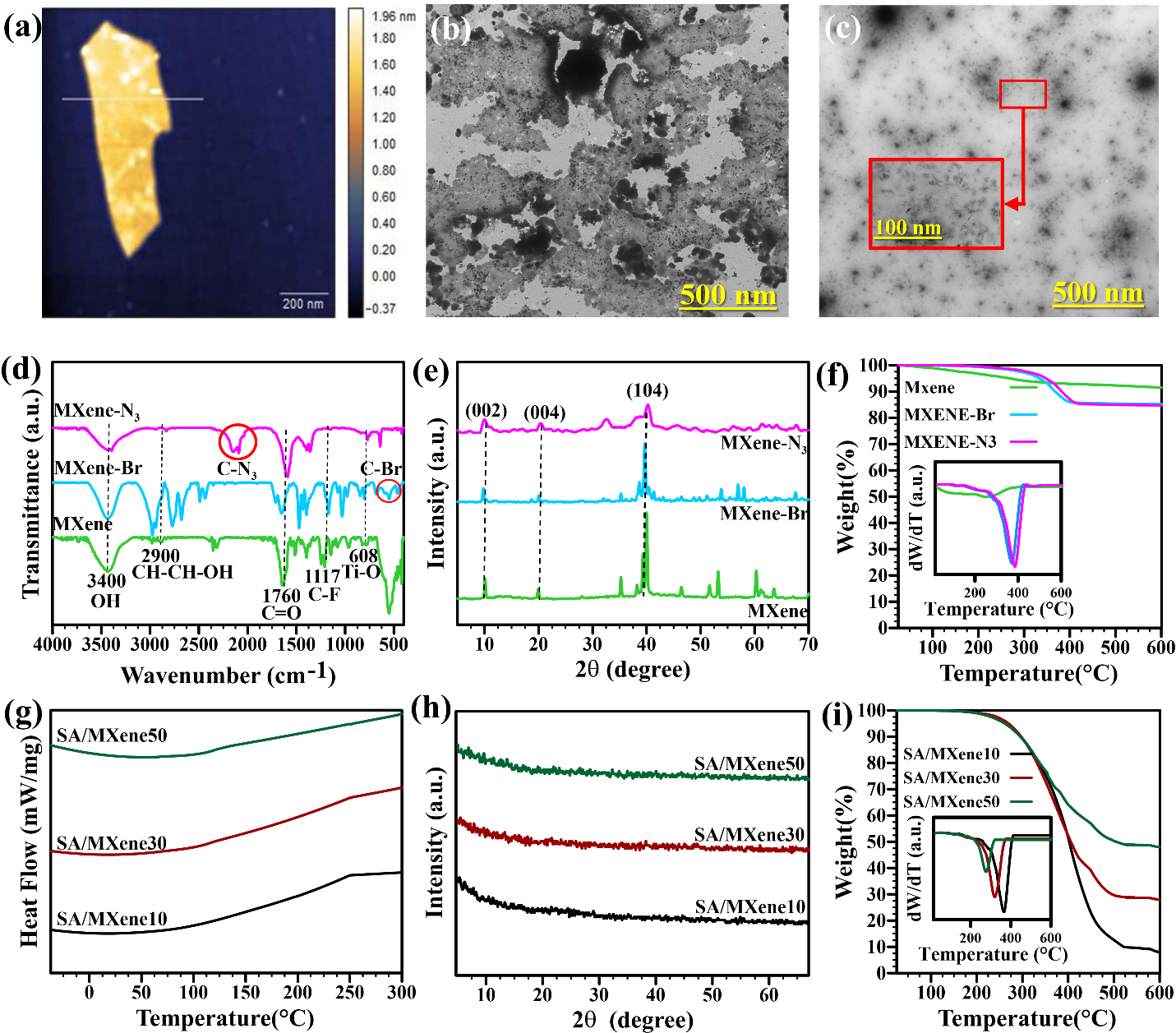


**Figure S1.** (a) AFM image of Ti_3_C_2_T_x_ MXene nanosheet [S2]; TEM images of (b) MXene-Br and (c) MXene-N_3_; FTIR (d), XRD (e), and TGA (f) analysis for MXene, MXene-Br, and MXene-N_3_ samples; DSC curves (g), XRD patterns (h), and TGA curves (i) of SA/MXene10, SA/MXene30, and SA/MXene50.

**Table S2.** Thermal properties of synthesized samples

| Sample | T_d,5%_ (°C) | T_f_ (°C) | T_max_ (°C) | Residual weight (%) |
| --- | --- | --- | --- | --- |
| MXene | No degradation peak, just smooth slope due to the water evaporation | | | |
| MXene-Br | 245.3 | 421.2 | 373.5 | 85.1 |
| MXene-N_3_ | 269.1 | 437.7 | 387.2 | 84.7 |
| SA/MXene10 | 238.9 | 414.7 | 367.4 | 9.4 |
| SA/MXene30 | 243.1 | 378.2 | 322.1 | 28.6 |
| SA/MXene50 | 249.4 | 324.5 | 227.0 | 48.1 |

T_d,5%_ is the initiation of degradation temperature, T_max_ is the maximum decomposition temperature, and T_f_ is the final decomposition temperature.

## **S2.2 Electrochemical properties**

The Polymer resistance (*R*_b_) and the interface resistance (*R*_s_) can be obtained by fitting the proper circuit model on the EIS data using ZSimpWin software (see **Figure S2**); and then for calculating, the ionic conductivity of a SPE, the value for *R*_b_ will be substituted in the **Equation S1**.

**
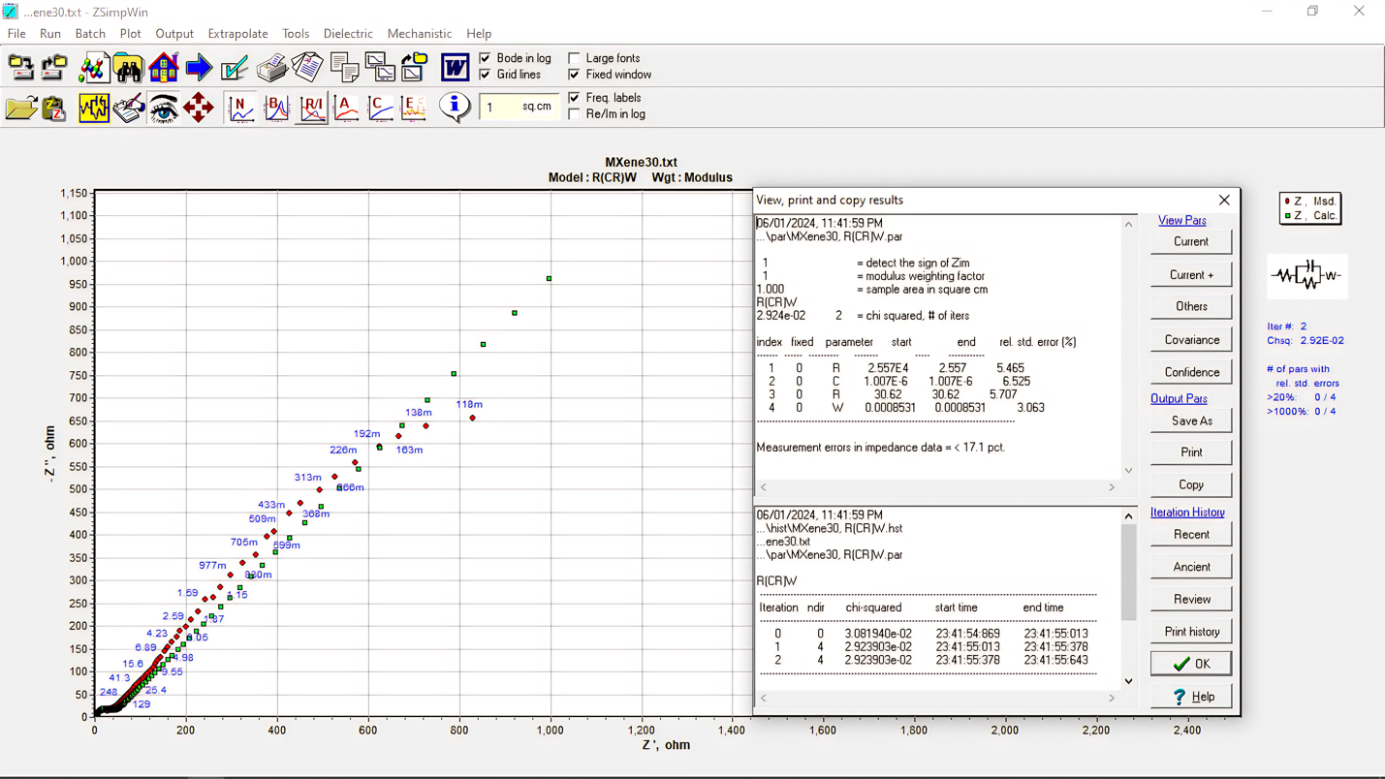
**

**Figure S2.** A sample of the fitted circuit model on to the EIS result; the green dots are the simulation and the red ones are the real data (the error of the fitted model is around 5%).

**
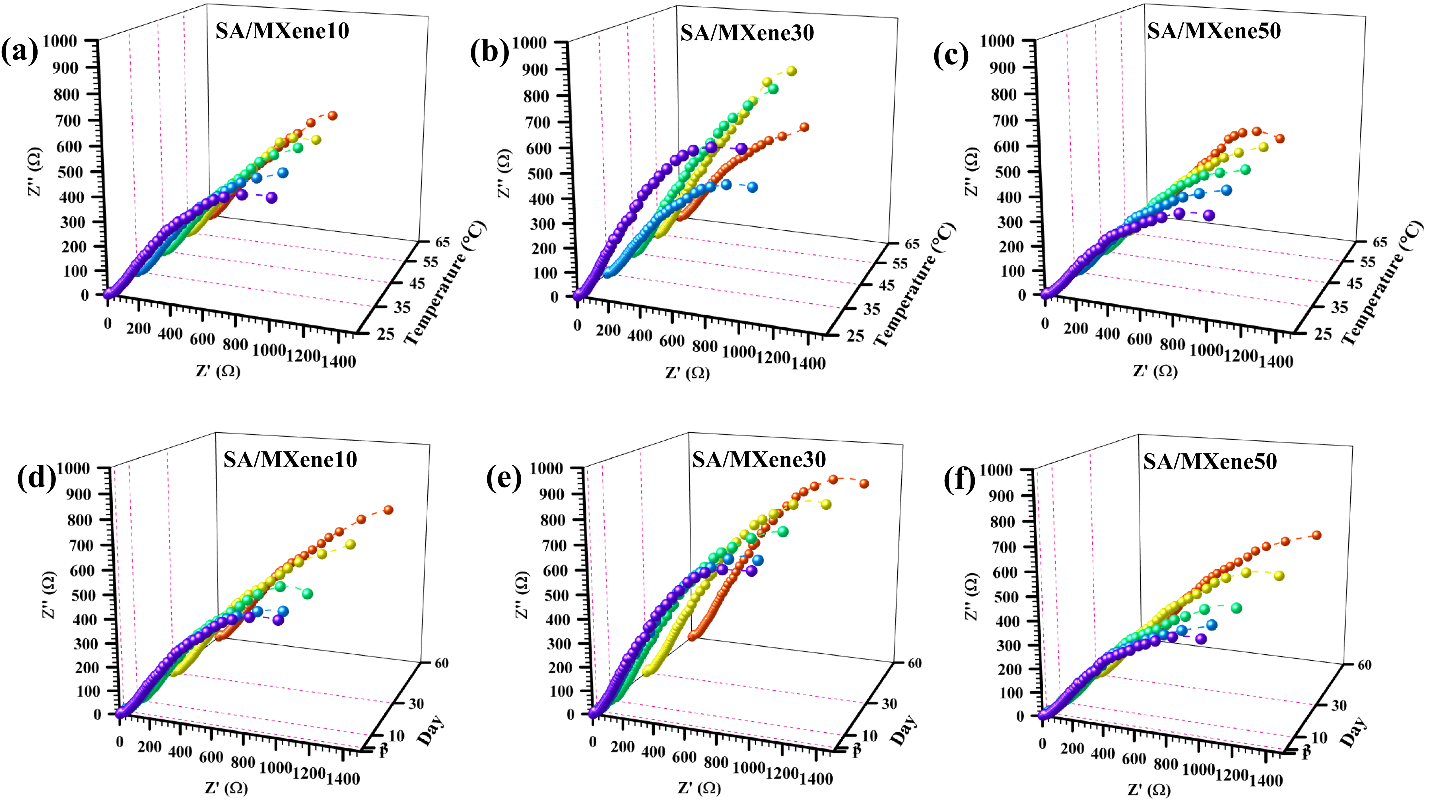
**

**Figure S3.** Impedance plots of SS/SPEs/SS at various temperatures (a to c) and days (d to f).

Arrhenius equation [6]:

$\sigma=Aexp(E_{a}/K_{B}T)$ (S3)

Here, $\sigma$ is ionic conductivity, *T* is temperature, k_B_ is the Boltzmann constant, *E*_a_ is the activation energy, and A is the pre-exponential factor.

**Table S3.** *E*_a_, and *A* values for the synthesized SPEs

| Polymer electrolyte | E_a_ (eV) | A (S cm^-1^) |
| --- | --- | --- |
| SA/MXene10 | 0.619 | 0.016032 |
| SA/MXene30 | 0.587 | 0.01584 |
| SA/MXene50 | 0.769 | 0.009452 |

The activation energy (*E*_a_) represents the minimum energy required for ions to migrate through the polymer matrix, reflecting the binding energy of ions to the polymer chains. Higher *E*_a_ values indicate stronger ion-polymer interactions, resulting in lower ionic conductivity. The polymer's structure, particularly the presence of amorphous regions and the availability of coordination sites (e.g., oxygen atoms), significantly influences *E*_a_. The prepared SPEs here, due to the complete amorphous structure, and high number of oxygen atoms in their structures, have such low *E*_a_, especially for SA/MXene30 electrolyte [7,8].

The pre-exponential factor *A* represents the frequency of successful ion movements or jumps within the polymer matrix, accounting for the number of available pathways and the vibrational frequency of ions. The higher *A* value indicates a greater number of effective ion conduction pathways, suggesting a more favorable structural arrangement for ion transport. The polymer's morphology, including the degree of amorphousness and the density of coordination sites, significantly influences *A*. In essence, *A* reflects the inherent efficiency of the ion conduction process in the material. The high *A* values observed for all SPEs, particularly SA/MXene30, indicate the high ability of these well-designed structures to facilitate efficient Li^+^ transport through the provision of suitable pathways [7-9].

**Table4.** Bulk resistance of initial polarization, final polarization, and ion transfer number values of prepared GPEs and SPEs

| Polymer electrolyte | R_s,0_ (Ω) | R_s,ss_ (Ω) | I_0_ (mA) | I_ss_ (mA) | t^+^ |
| --- | --- | --- | --- | --- | --- |
| SA/MXene10 | 2.87 | 3.55 | 0.290 | 0.235 | 0.81 |
| SA/MXene30 | 2.55 | 2.82 | 0.343 | 0.312 | 0.91 |
| SA/MXene50 | 3.14 | 4.02 | 0.272 | 0.214 | 0.78 |

**
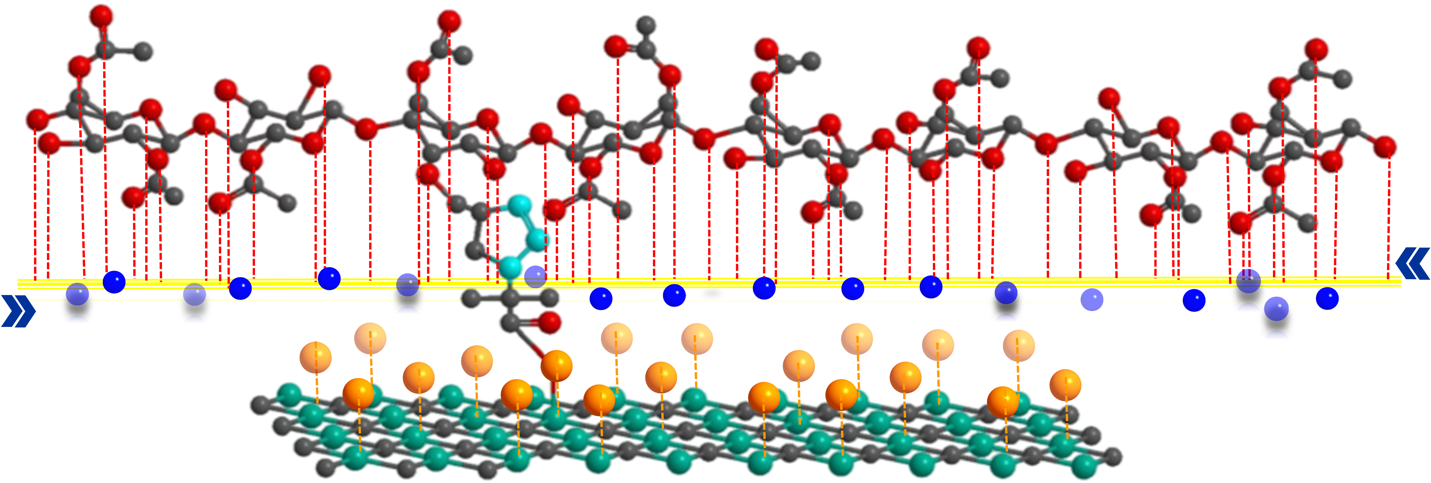
**

**Figure S4.** The schematic of the ion pathway created by oxygen atoms of SA and the stabilization of X^-^ through the MX-QDs (red dash line represents coordination between the oxygen atoms and Li^+^ ions)

**Table S5.** Values for ionic conductivity of synthesized SPEs; capacity, Columbic efficiency (CE), and retained capacity (RC) for prepared ASSLIBs

| Polymer electrolyte | $\boldsymbol{\sigma}$ (mS cm^-1^) | CE (0.1C)-1^st^ cycle | Capacity (mAh g^-1^) at 0.1C current density | RC (0.1) | | | | |
| --- | --- | --- | --- | --- | --- | --- | --- | --- |
|  |  |  |  | **20** | **50** | **100** | **500** | **1000** |
| SA/MXene10 | 11.3 | 0.89 | 172.01 | 156.39 | 149.92 | 149.79 | 149.30 | 148.84 |
| SA/MXene30 | 14.8 | 0.90 | 178.38 | 150.92 | 150.13 | 150.00 | 149.51 | 149.04 |
| SA/MXene50 | 10.8 | 0.93 | 171.37 | 156.05 | 155.23 | 155.11 | 154.59 | 154.11 |

**References**

[S1] Hadad, S., Hamrahjoo, M., Dehghani, E., Salami-Kalajahi, M., Eliseeva, S. N., Moghaddam, A. R., & Roghani-Mamaqani, H., Starch acetate and carboxymethyl starch as green and sustainable polymer electrolytes for high-performance lithium-ion batteries, 2022, Appl. Energy, 324, 119767, https://doi.org/10.1016/j.apenergy.2022.119767

[S2] Zarshenas, K., Dou, H., Habibpour, S., Yu, A., & Chen, Z., Thin Film Polyamide Nanocomposite Membrane Decorated by Polyphenol-Assisted Ti3C2Tx MXene Nanosheets for Reverse Osmosis, 2021, ACS Appl. Mater. Interfaces, 14(1), 1838-1849, https://doi.org/10.1021/acsami.1c16229

[S3] Baskoro, F., Wong, H. Q., & Yen, H. J., Strategic structural design of a gel polymer electrolyte toward a high efficiency lithium-ion battery, 2019, ACS Appl. Energy Mater., 2(6), 3937-3971, https://doi.org/10.1021/acsaem.9b00295

[S4] Tian, Z., & Kim, D., Solid electrolyte membranes prepared from poly (arylene ether sulfone)-g-poly (ethylene glycol) with various functional end groups for lithium-ion battery, 2021, J. Membr. Sci., 621, 119023, https://doi.org/10.1016/j.memsci.2020.119023

[S5] Feng, A., Hou, T., Jia, Z., Zhang, Y., Zhang, F., & Wu, G., Preparation and characterization of epoxy resin filled with Ti3C2Tx MXene nanosheets with excellent electric conductivity, 2020, Nanomaterials, 10(1), 162, https://doi.org/10.3390/nano10010162

[S6] Bocharova, V., & Sokolov, A. P., Perspectives for polymer electrolytes: a view from fundamentals of ionic conductivity, 2020, Macromolecules, 53(11), 4141-4157, https://doi.org/10.1021/acs.macromol.9b02742

[S7] Wang, L., He, Y., & Xin, H. L., Transition from Vogel-Fulcher-Tammann to Arrhenius Ion-Conducting Behavior in Poly (Ethyl Acrylate)-Based Solid Polymer Electrolytes via Succinonitrile Plasticizer Addition, 2023, J. Electrochem. Soc., 170(9), 090525, https://doi.org/10.1149/1945-7111/acf881

[S8] Lin, Z., Guo, X., Wang, Z., Wang, B., He, S., O'Dell, L. A., Huang, J., Li, H., Yu, H., & Chen, L., A wide-temperature superior ionic conductive polymer electrolyte for lithium metal battery, 2020, Nano Energy, 73, 104786, https://doi.org/10.1016/j.nanoen.2020.104786

[S9] Gainaru, C., Kumar, R., Popov, I., Rahman, M. A., Lehmann, M., Stacy, E., Bocharova, S., Saito, T., Schweizer, K., & Sokolov, A. P., Mechanisms Controlling the Energy Barrier for Ion Hopping in Polymer Electrolytes, 2023, Macromolecules, 56(15), 6051-6059, https://doi.org/10.1021/acs.macromol.3c00879
